# Temporal Separation of Aggregation and Ubiquitination during Early Inclusion Formation in Transgenic Mice Carrying the Huntington’s Disease Mutation

**DOI:** 10.1371/journal.pone.0041450

**Published:** 2012-07-24

**Authors:** Belvin Gong, Catherine Kielar, A. Jennifer Morton

**Affiliations:** 1 Department of Pharmacology, University of Cambridge, Cambridge, United Kingdom; 2 UC Davis/NIH NeuroMab Facility, Department of Neurobiology, Physiology and Behavior, University of California Davis, Davis, California, United States of America; Mount Sinai School of Medicine, United States of America

## Abstract

Abnormal insoluble ubiqitinated protein aggregates are found in the brains of Huntington’s disease (HD) patients and in mice transgenic for the *HTT* mutation. Here, we describe the earliest stages of visible NII formation in brains of R6/2 mice killed between 2 and 6 weeks of age. We found that huntingtin-positive aggregates formed rapidly (within 24–48 hours) in a spatiotemporal manner similar to that we described previously for ubiquitinated inclusions. However, in most neurons, aggregates are not ubiquitinated when they first form. It has always been assumed that mutant huntingtin is recognised as ‘foreign’ and consequently ubiquitinated and targeted for degradation by the ubiquitin-proteasome system pathway. Our data, however, suggest that aggregation and ubiquitination are separate processes, and that mutant huntingtin fragment is not recognized as ‘abnormal’ by the ubiquitin-proteasome system before aggregation. Rather, mutant Htt appears to aggregate before it is ubiquitinated, and then either aggregated huntingtin is ubiquitinated or ubiquitinated proteins are recruited into aggregates. Our findings have significant implications for the role of the ubiquitin-proteasome system in the formation of aggregates, as they suggest that this system is not involved until after the first aggregates form.

## Introduction

Huntington’s disease (HD) is one of a family of progressive genetic neurodegenerative disorders caused by the pathological expansion of a CAG repeat in the *HTT* disease gene that encodes the protein huntingtin (Htt) [1]. The mechanism by which the CAG repeat expansion causes HD is not known. However, the discovery of neuronal intranuclear inclusions (NIIs) in the brains of mice transgenic for a protein fragment carrying the *HTT* mutation [2] and subsequently in brains of HD patients [2], [3] has triggered a great deal of interest in both the mechanisms of inclusion formation and their potentially pathogenic role. The importance of inclusion pathology is not restricted to HD, since inclusions are present in brains of patients with other polyglutamine diseases and all mouse models thus far examined (for review, see [5]). Nevertheless, the role of inclusions in HD pathology is not only unclear, but is also hotly debated (for review see [6]). There is evidence suggesting that they may be neurotoxic ([7], [8], [9], [10], [11], [12], [13], [14], neuroprotective [15], [16], [17], [18], [19], [20], [21], [22], [23], [24] or both, depending on when and where they form [25], [22].

Despite the debate about their role, there is no doubt that inclusions are a clear histopathological marker of the disease [26]. Inclusions are not found in neurologically normal subjects, but are found throughout the HD brain, particularly in striatum (STR) and cortex (CTX), the brain regions most affected in HD [3], [4], [16], [27], [28]. NIIs are defined as abnormal ubiquitinated aggregates of proteins, predominantly huntingtin and/or fragments of huntingtin and ubiquitin, although a number of other proteins have been found associated with inclusions in transgenic mouse and cell models [29], [30] and human brains [31]. Importantly, it has always been assumed that mutant huntingtin is recognised as ‘foreign’ and consequently ubiquitinated and targeted for degradation by the ubiquitin-proteasome system pathway, because (1) a mutation in the gene coding for huntingtin causes HD, (2) mutant huntingtin is found in neuronal intranuclear inclusions, (3) neuronal nuclear inclusions are ubiquitinated, (4) the ubiquitin-proteasome pathway is responsible for recognising and disposing of abnormal proteins and (5) proteasome fragments are associated with NIIs.

To understand the role of NIIs in HD pathology, it would help if we knew what relationship exists between the appearance of inclusions, their ubiquitination and the onset of neuronal dysfunction. Here, we focused on the first stage of inclusion formation. We used juvenile R6/2 mice to study *ex vivo* the processes of Htt aggregation and inclusion formation. R6/2 mice show progressive neurological impairments [32], [33], [34], [35] and the appearance of ubiquitinated inclusions precedes the appearance of measurable behavioral (motor and cognitive) phenotypic changes [25] and happens at around the same time as abnormalities in synaptic plasticity [36] and early changes in brain markers [37] are first seen.

We performed an extensive and comparative immunohistochemical analysis of Htt aggregation and inclusion ubiquitination to pinpoint both the order of appearance and the regional location of aggregates in R6/2 brain. For this, we used the MW8 antibody that is specific for the aggregated conformation of mutant Htt protein [38], along with anti-ubiquitin antibodies. We showed that visible Htt-immunopositive aggregates are present in R6/2 brain as early as 2 weeks of age and appear in a region specific manner throughout the brain over the next few weeks. Notably, individual Htt-positive aggregates formed very rapidly, within the interval of a single day in most brain regions. Our data suggest that mutant Htt aggregation occurs rapidly and is then either ubiquitinated or other ubiquitinated proteins (that may include Htt itself) are recruited into inclusions.

## Methods

### R6/2 Mice

Mice were taken from a colony of R6/2 mice established in the Department of Pharmacology, University of Cambridge, as previously described [33]. The line was maintained by back-crossing transgenic males onto female CBA×C57BL/6 F1 mice. All mice used in this study were taken from the 21^st^ and the 23^rd^ generations of back-crosses. The CAG repeat lengths of transgenic mice used were measured and the mean repeat length was calculated as 188±1 (N = 122; see genotyping section for more information). All mice were housed with a 12-hour light/dark cycle in a temperature- and humidity-controlled environment. Food (mash and dry food) and water were available *ad libitum.*

Mice used for the earliest time-points (4 weeks of age and younger) were not weaned. Older mice were weaned, separated by gender and housed in groups of mixed genotype at 4 weeks.

### Time-Course Tissue Collection Scheme

We wanted to ensure that for all time-points we had at least 3 animals of each genotype from three different litters, and that for each animal we had one littermate that was exactly 24 hours younger and one that was 24 hours older (except for the 14 day timepoint where we only needed an older littermate). For time-course time-points between 14 and 28 days of age, brain tissue was collected at intervals of 24 hours. This interval was chosen because *in vitro,* aggregation is observed within this timeframe. Since most of the early time-course time-points used in this study preceded the age at which we were able to take samples for genotyping (2.5–3 weeks of age), a tissue collection ‘scheme’ was used for mice up to 25 days as follows. We used only litters that contained 9–12 pups. We did not distinguish between the sexes. Three or four mice were randomly selected from each litter to be killed at the same time of day on each of 3 consecutive days. Thus, any given mouse, on any given day, had at least one WT and one R6/2 littermate killed 24 hours before or 24 hours after it had been killed. This allowed us to compare regional appearance of inclusions in littermates at 24 hourly intervals. Performing the time-course in this way allowed us to examine variability between animals killed at specific time-points as well as between littermates exactly 24 hours apart. R6/2 and WT mice killed at ages greater than 25 days (4, 5, 6, 9 and 12 weeks) were killed according to their genotypes (N = 4 R6/2 and 3 WT littermates at each age).

Animals were genotyped to ascertain the distribution of R6/2 and WT within these groups. We used 12 litters (122 mice) for tissue collection for time course points up to 25 days, and a total of 35 mice for the other time points. All brains were dissected, frozen on powdered dry ice and stored at −80°C until use as previously described (Morton *et al*., 2000).

### Genotyping

Genotyping was performed by PCR from tail snips taken at 3 weeks of age and CAG repeat lengths were measured by Laragen (USA). All CAG repeat numbers reported are those determined directly by Genemapper software (note that the CAG repeat number measured by GeneMapper differs from that measured by sequencing). To convert the CAG repeat numbers determined by GeneMapper technique to the CAG repeat number determined by sequencing technique (which more closely represents the true CAG repeat number) the following formula needs to be applied: SEQ CAG no. (true CAG no.)  = 1.0427 * GM CAG no. +1.1695; personal communication, Dr J. Li, Laragen).

### Immunohistochemistry

The antibody staining procedure was performed essentially as previously described [25]. Antibodies used were either a rabbit polyclonal antibody raised against poly-ubiquitin (1∶2000; Dako, Denmark) or a mouse monoclonal antibody MW8 raised against Htt (1∶2000; kind gift of Dr. Paul Patterson, Caltech, CA, USA). The MW panel of antibodies was raised against synthetic peptides of polyglutamine disease protein fragments with expanded repeats [38]. MW8 was raised against the first exon of mutant Htt with antigenic boosts of aggregated Htt protein [38]. MW8 recognises the aggregated conformation of mutant Htt, and epitope-mapping studies reveal that it recognises the C-terminal portion of exon 1, specifically the sequence AVAEEPLHRPK [38]. This sequence shows no known homology to any protein other than Htt (BLAST searches). MW8 has been reported to label nuclear inclusions in R6/2 brain exclusively (although in our hands it also stains neuropil aggregates, see below). It shows no staining above background in WT animals (this study; [38]). Horseradish peroxidase (HRP)-conjugated secondary antibodies were used to label bound primary antibodies (1∶2000; Vector, Peterborough, UK). Importantly, the MW8 staining here was obtained with standard histological techniques and without assistance from antigen retrieval methods that are required to ‘reveal’ aggregates that were not seen before (e.g. formic acid treatment [39], [40]).

Cryosections of fresh frozen R6/2 and WT brain tissue were cut at 30 µm thickness onto gelatin-coated glass slides and processed for immunohistochemical staining. Sections were peroxidase-inactivated for 15 min at room temperature (20% v/v methanol, 0.2% Triton X-100 and 1% v/v hydrogen peroxide in phosphate-buffered saline (PBS)) and incubated in block solution (3% deer serum, 0.2% Triton X-100 and 0.1% sodium azide in PBS) for at least 3 hours at room temperature. At least one run of staining included sections from all of the animals used in the study and all of the sections were incubated in the same solution. Parallel sections were incubated with primary antibodies diluted in block solution at 4°C for a week, extensively washed and incubated with secondary antibodies diluted into wash solution at 4°C overnight. We used parallel sections rather than double staining to be sure that there was no false positives. Parallel sections allowed us to repeat staining on negative sections. Immunoreactivity was visualised using diaminobenzidine (Sigma, Poole, UK). All of the staining was done in parallel, timed to within 5 seconds over a 10 minute incubation period, in the same solution. A confirmation series of staining was done with one slide (a series of sections) from every animal in the study developed together. A slide with parallel sections from each brain was stained with 1% cresyl violet acetate solution in water (CV), mounted in Histoclear (CellPath, Newtown, UK) to aid neuroanatomical identification of brain regions. All immunostained slides were counterstained with Hoechst 33258 dye (5 µg/ml in PBS for 30 min) to label nuclei, mounted in glycerol jelly, covered with a coverslip and stored in the dark at room temperature until analysis.

**Figure 1:**
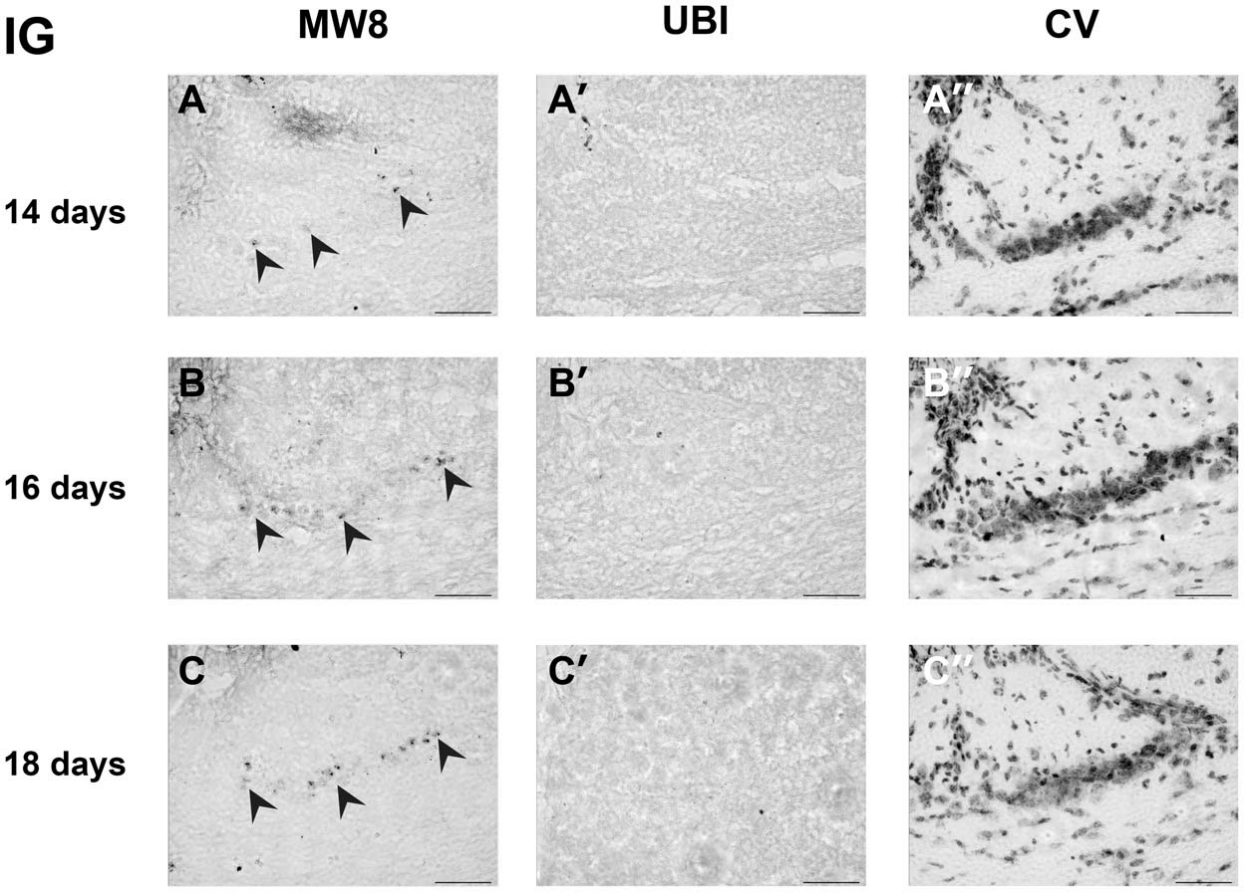
Aggregate appearance in indusium griseum(IG). Htt aggregates visualised by staining with the MW8 antibody were present in adjacent sections for IG of R6/2 brains as early as 14 days of age (A, black arrowheads) and increased in number with time (B, C; black arrowheads). Staining with ubiquitin antibody did not reveal the presence of inclusions in any region at these time points (A’–C’). Cell distribution in this region was visualised with a cresyl violet (CV) stain (A”–C”). Scale bar  = 50 µm.

### Light and Fluorescence Microscopy

We have shown previously that ubiquitinated NIIs appear first in juvenile R6/2 brain in the indusium griseum (IG), where they are visible by 3 weeks of age. Then, between 3 and 4.5 weeks of age NIIs appear in the CA1 region of hippocampus (Hf), layers II/III and V of frontal and parietal CTX, and STR [25]. In the current study, we again focused on these brain regions, because the different cell types in them are easily identified by their anatomical location.

**Figure 2:**
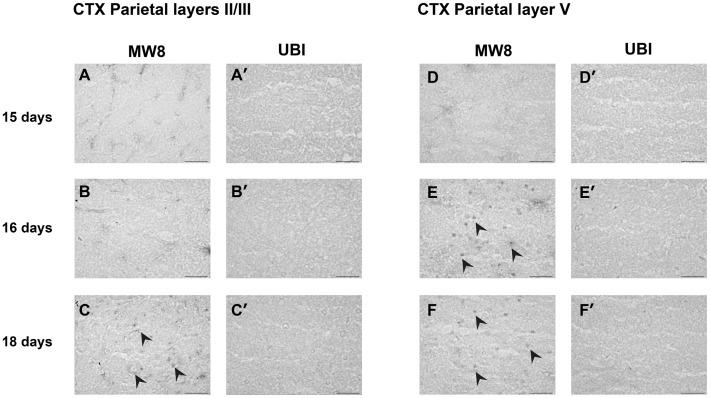
Aggregate appearance in parietal cortex (CTX). Htt aggregates visualised by staining with the MW8 antibody were present in the CTX of R6/2 brains at 16 days in layer V (E, F; black arrowheads) and at 18 days in layer II/III (C, black arrowheads). Staining with ubiquitin antibody did not reveal the presence of inclusions in any region at these time points (A’–F’). Scale bar  = 50 µm.

**Figure 3:**
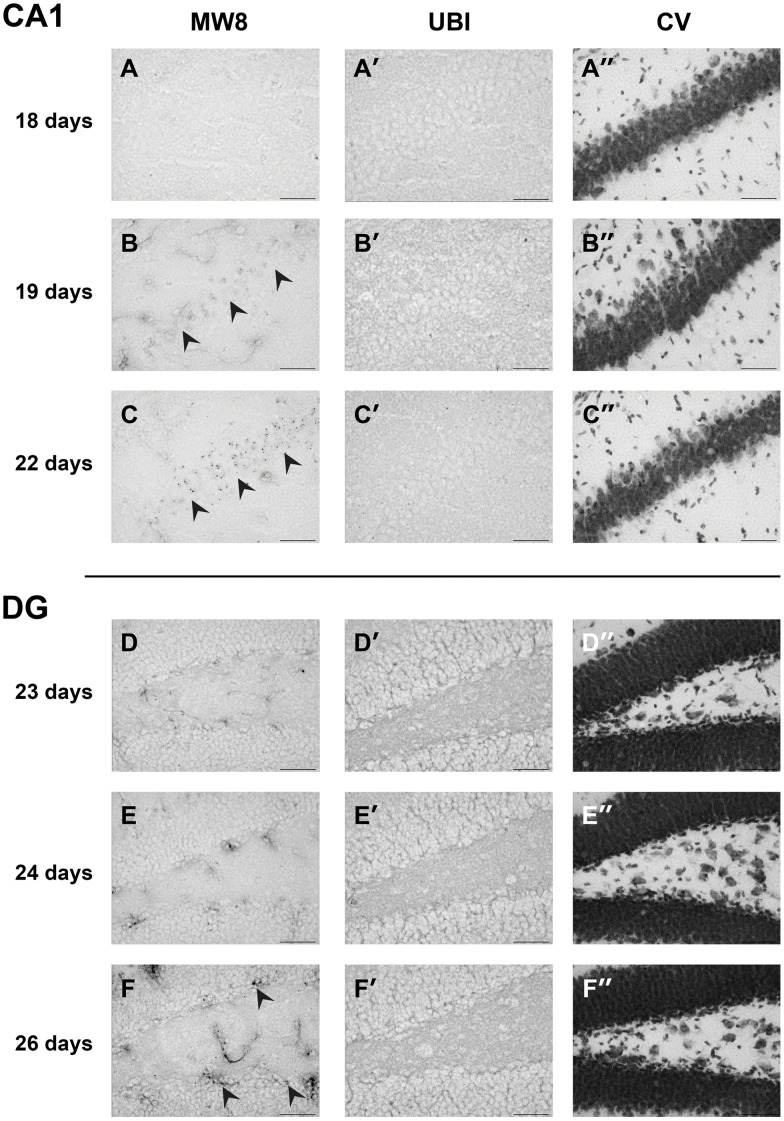
Aggregate appearance in CA1 and dentate gyrus (DG). In R6/2 hippocampus, Htt aggregates visualised by staining with the MW8 antibody could not be seen at 18 days of age but could be found in many neurons at 19 days in the CA1 (B, black arrowheads) and increased in number with time (C, black arrowheads). Cell distribution in this region is shown in a parallel section showed with a cresyl violet stain (A”–C”). In the DG, MW8-positive aggregates were absent up to 24 days, but were present at 26 days (F, black arrowheads). Staining with ubiquitin antibody did not reveal the presence of inclusions in any region at these time points (A’–F’). Cell distribution in both regions was visualised with a cresyl violet (CV) stain (D”–F”). Scale bar  = 50 µm.

Slides were analysed using an inverted fluorescence Nikon Eclipse TE-2000 microscope (Nikon, Surrey, UK). Hoechst 33342 staining of nuclei was visualised with UV light excitation and a DAPI filter. Photomicrographs were taken at X40 and X100 (under oil immersion) objective magnification with the same time exposure settings using the Nikon Still Camera DXM1200 and the Nikon Lucia software system (Nikon, Surrey, UK).

**Figure 4:**
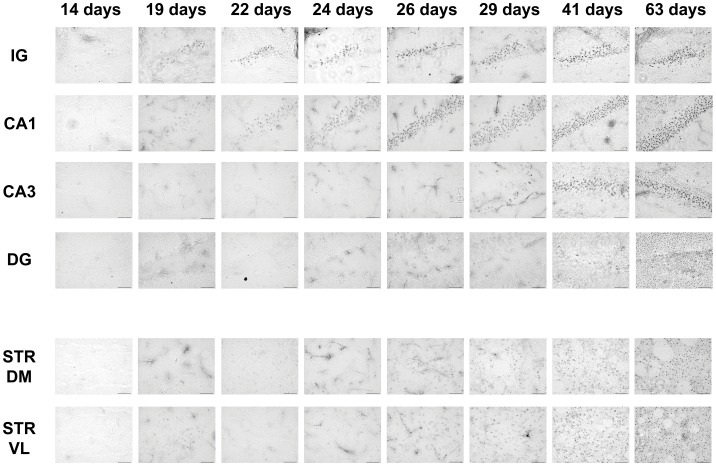
Aggregate appearance in different brain regions over time. Htt aggregates visualised by staining with the MW8 antibody (shown here at 14, 19, 22, 24, 26, 29, 41 and 63 days) in indisium griseum (IG), CA1, CA3, dentate gyrus (DG) and dorso-medial and ventro-lateral striatum (STR DM and STR VL) of R6/2 brains. A few aggregates were visualised in the IG as early as 14 days of age. In the hippocampus, inclusions were present at 19 days in CA1, whereas in the CA3 a few aggregates were visible at 29 days of age. In the dentate gyrus, MW8-positive aggregates were present at 26 days. In the striatum, Htt aggregates could be seen at 26 days of age in STR DM and STR VL. The number of aggregates in all the regions increased with time from the day of appearance. Scale bar  = 50 µm.

### Aggregates and Inclusions

In previous studies of protein aggregation in HD brain, the terms ‘aggregate’ and ‘inclusion’ have been used interchangeably to identify ubiquitinated deposits of proteins in human and mouse HD brain. This has not caused difficulties in discussions of ‘inclusions’ for two main reasons. Firstly, ubiquitin antibody staining is generally accepted as the benchmark marker for identifying inclusions. Secondly, few research groups have made detailed studies of the difference between ubiquitin-positive inclusions and Htt-positive aggregates. However, the use of MW8 in the course of this study showed it was possible to clearly distinguish aggregates of Htt from aggregates with the classical morphological appearance of ubiquitinated ‘inclusions’. Therefore, we have used the term ‘inclusion’ only when referring to an aggregation of protein in the nucleus that is ubiquitinated, such as were classically described in human and mouse HD brain [2], [25]. It is also important to stress that this study did not include microaggregate foci and focused solely on aggregates.

**Figure 5:**
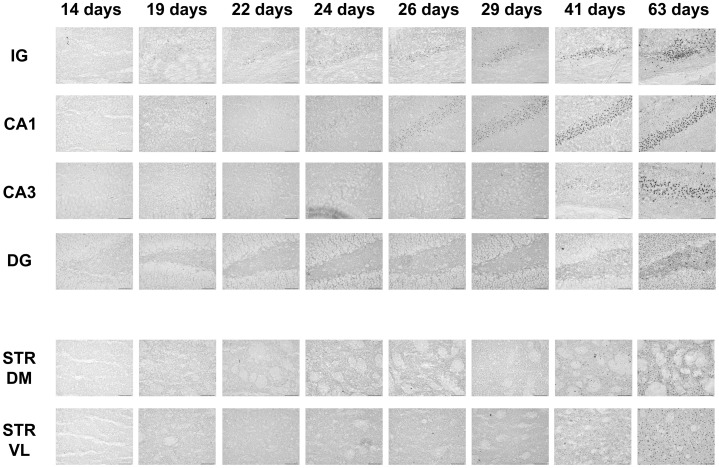
Inclusion appearance in different brain regions over time. Ubiquitinated Htt aggregates visualised by staining with the ubiquitin antibody (shown here at 14, 19, 22, 24, 26, 29, 41 and 63 days) in IG, CA1, CA3, DG and STR of R6/2 brains. A few inclusions are visible in the IG at 19 days of age. In the hippocampus, aggregates were present at 24 days in CA1, whereas in the CA3 a few aggregates were visible at 41 days of age. In the DG, a few ubiquitin-positive inclusions were present at 41 days. In the striatum, inclusions could be setected at 41 days of age in STR DM and STR VL. The number of aggregates in all the regions increased with time from the day of appearance. Scale bar  = 50 µm.

### Aggregated Huntingtin

For convenience, throughout this paper, we describe the proteins that are visualised by MW8 as ‘aggregated mutant huntingtin’. This is not strictly accurate, since the transgene includes only the first exon of the HD gene. Thus, the major protein species that is found in inclusions in R6/2 mice is not full length huntingtin, but rather a mutant fragment of huntingtin with a pathological length polyglutamine repeat.

## Results

### Htt Aggregation is a Rapid and Early Event

By comparing littermates that were killed exactly 24 hours apart, we were able to determine the age at which Htt aggregates first became visible at a light level. MW8-positive aggregates were already present in the IG of R6/2 brains by 14 days of age (Fig. 1), but were not visible in any other brain region. Thus the aggregates in IG were used as a reliable internal positive control for the MW8 antibody staining.

**Figure 6:**
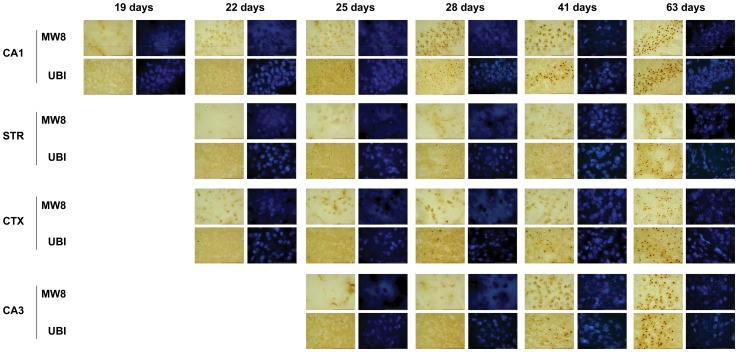
Thetiming of ubiquitination is dependent of cell soma size in R6/2 neurons. Regardless of whether or not huntingtin aggregates (visualized by staining with MW8 and ubiquitin antibodies) formed early or late, aggregates that formed in cells with small (DG neurons) and medium-sized (CA1 and STR) soma were found ubiquitinated within 2 to 3 days of initial aggregation. By contrast, there was typically a more than 3 days lag between initial aggregate formation and inclusion ubiquitination in cells with large soma (CA3). Scale bar  = 50 µm.

In many cells, Htt aggregates that could be visualised by staining with MW8 appeared almost overnight. For example, in the parietal cortex, MW8-positive aggregates were absent from all layers at 15 days (Fig. 2A, A’, D and D’). Aggregates were present at 16 days in layer V (Fig. 2E and E’), but not in layer II/III (Fig. 2B and B’). By 18 days aggregates were also present in layer II/III (Fig. 2C, C’). Similarly, in CA1 cells of the hippocampus, Htt aggregates could not be seen at 18 days of age (data not shown) but were visible as diffuse aggregates in many neurons at 19 days (Fig. 3B, B’ and B”) and clearly present by 22d (Fig. 3C, C’ and C”).

**Figure 7:**
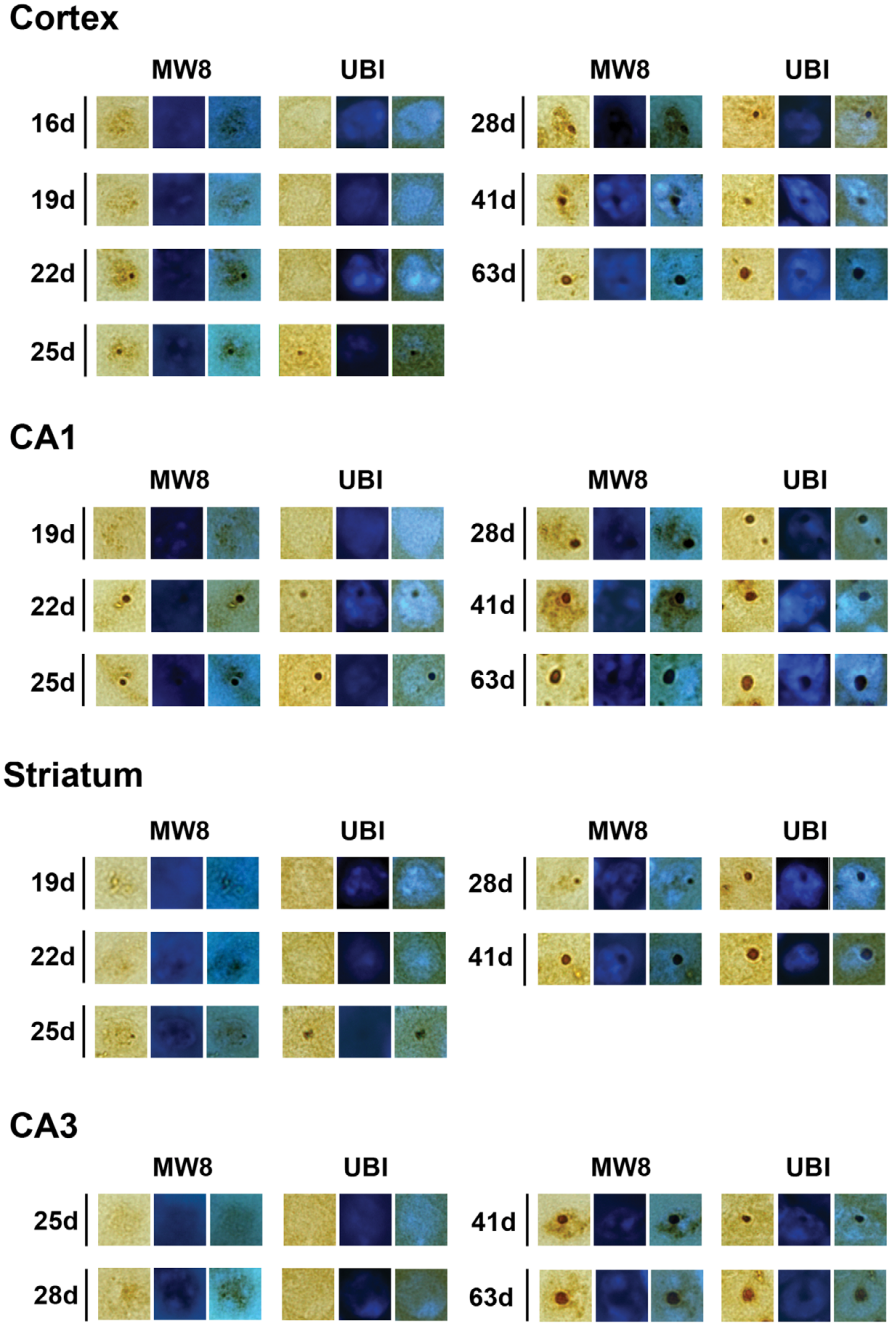
Comparison of MW8 and ubiquitin staining patterns. Higher magnification microscopy analysis was used in order to compare MW8 and ubiquitin staining patterns in single R6/2 neurons. In CA1, CA3 and cortex, MW8 labelling could be observed in cells before any aggregates were visible, a phenomenon that was not apparent with ubiquitin staining. The initial morphologies of MW8-positive aggregates were different from ubiquitin-positive inclusions, in that only nucleation centres of the aggregates were ubiquitinated and the MW8-immunolabelled Htt protein that was not localised to the nucleation centre did not appear to be ubiquitinated. In contrast, aggregates with clear nucleation centres were visible by 25 days in the striatum. And at the same time, larger punctate ubiquitin-labelled inclusions were already visible in STR neurones. Ubiquitinated inclusions were much bigger than MW8-positive aggregates and did not change much in size, even though Htt aggregates got bigger with time.

Both the number of cells with aggregates and the levels of immunostaining intensity varied markedly in different regions of the same brain, suggesting that Htt aggregation is timed according to the identity of the cell population. However, aggregate formation appeared to follow a similar pattern in neurons of a single class. For example in pyramidal cells in the CA1 region of the hippocampus, MW8-positive aggregates first appeared with a diffuse morphology on day 19 (Fig. 3 and Fig. 4). Over the next few days, the Htt aggregates changed from being amorphous to having clear nucleation centres that became visible at 22 days (Fig. 3 and Fig. 4). This pattern was typical of all CA1 neurons. By contrast, in the dentate gyrus (DG) of the hippocampus Htt aggregates were first seen at 26 days (Fig. 3, Fig. 4), many days after they were first observed in CA1 (Fig. 3 and Fig. 4). Interestingly, it is not just the timing that varies, but also the morphology of aggregate formation. Htt aggregates in DG neurons had a more punctate morphology than those first seen in CA1 cells (Fig. 3), and they continued to grow with time (Fig. 4) until they reached the stage where MW8-stained aggregates were of similar appearance to the nucleation centres described by Chen *et al*., [41]. By contrast, aggregates in cortical neurons were similar to those that formed in CA1 neurons, beginning as diffuse deposits in nuclei and then clustered into MW8-positive aggregates with nucleated centres.

### Aggregate Formation in different Brain Regions is Differentially, but Precisely, Timed

There was a defined and consistent order to the organisation and timing of first appearance of Htt aggregates in R6/2 brains. As mentioned above, in the IG Htt aggregates were already present at 14 days of age (Fig. 1). Subsequently, Htt aggregates appeared (by 19 days of age), in the CA1 region of the Hf (Fig. 4) and the deeper layers of parietal, frontal and cingulate CTX. Aggregates also began to form in cells in layer II/III of parietal CTX (but not other parts of the CTX) at this time (Fig. 2). Between 19 and 22 days, aggregates in IG and CA1 grew larger (Fig. 3). By 22 days, almost all (>80%) cells in the deep layers of parietal CTX had visible aggregates that were MW8-positive (Fig. 2). Over the following few days marked increases in aggregate size and frequency of appearance in brain regions already containing aggregates were observed, while Htt aggregates just began to form in the DG and in STR by 26 days (Fig. 4). Finally, CA3 neurones began to form visible Htt-positive aggregates by 29 days (Fig. 4). By 41 days, the majority of cells contained aggregates of Htt protein in dense nuclear foci (Fig. 4). The exceptions were DG and CA3 cells, in which progression of aggregate formation was much slower.

### Htt Aggregation Precedes Ubiquitination

In all R6/2 brain regions studied, the appearance of Htt-positive aggregates preceded inclusion ubiquitination. At 14 days of age, there were no ubiquitinated inclusions in any brain region, which reproduces previous observations that no NIIs could be found in any brain regions of R6/2 mice at that age [25], [42]. The appearance of ubiquitinated inclusions occurred first in the IG by 19 days (Fig. 5). Ubiquitinated inclusions in other brain regions were observed at 24 days, when neurones in the CA1 region of the Hf began to show round amorphous ubiquitin-stained inclusions (Fig. 5). Over the next few days, there was a marked increase in the size and frequency of ubiquitinated inclusions in the IG as well as CA1. By 26 days, many IG and CA1 aggregates were ubiquitinated (Fig. 5). By 29 days, although Htt aggregates were present in neurons of the STR, DG and CA3 of the Hf, none of them were ubiquitinated (Fig. 5). At 41 days (nearly 6 weeks), aggregates in many brain regions were ubiqutinated, and continued to grow bigger, as seen previously [25]. They appeared to have reached a stable density in the IG and in CA1 (Fig. 5). In addition, by that age, ubiquitinated aggregates were present in the CA3. The only brain region in which cells did not have ubiqutinated inclusions by 41 days of age was the DG (Fig. 5). These observations are generally consistent with previous studies, where ubiqutinated NIIs were seen in DG only at 7 weeks of age and beyond [25]. By 63 days (9 weeks), when all neurones studied had aggregates (see Fig. 5), most were mature, ubiquitinated inclusions.

### Patterns of Ubiquitination Depend on Cell Soma Size

Previous observations have shown that increases in NII size are not due to increases in the sizes of nuclei [25], however the timing of initial aggregation formation with regard to the sizes of nuclei has not been studied until now. Here we compared cells in specific brain regions to see if a relationship existed between cell soma size, the propensity to form Htt aggregates and the time-course of ubiquitination. Neurons were classified as having small, medium or large soma. Granule cells of the DG were classified as ‘small’, CA1 pyramidal neurons and medium spiny striatal neurons were classified as ‘medium-sized’ and CA3 and cortical pyramidal neurons were classified as having ‘large’ soma. A direct comparison of MW8 staining patterns in cells from these brain regions showed that there was no correlation between the timing of aggregate formation and cell soma size (Fig. 6). For example, cells with medium-sized soma can form Htt aggregates as early as 22 days (CA1 neurons) or as late as 28 days of age (STR neurons). Likewise, cells with large soma can form aggregates as early as 19 days (neurons in parietal CTX) or as late as 28 days of age (CA3 neurons) (Fig. 6). However, timing of ubiquitination appeared to be related to cell soma size. Regardless of whether or not huntingtin aggregates formed early or late, aggregates that formed in cells with small and medium-sized soma were found ubiquitinated within 2–3 days of initial aggregation (Fig. 6). By contrast, there was usually lag of >3 days between initial aggregate formation and inclusion ubiquitination in cells with large soma (Fig. 6). For example, although aggregates formed early in cortical cells (19 days), ubiquitinated inclusions did not typically appear until after 22 days. In CA3 neurons, although Htt aggregates appeared by 29 days (Fig. 6), not all aggregates had been ubiquitinated before the end of the period over which we conducted 24-hour interval time-course study (that is, in CA3 neurons, some aggregates were ubiquitinated later than 41 days).

### Structural Investigation of Aggregate and Inclusion Formation

In order to compare MW8 and ubiquitin staining patterns in single R6/2 neurons in the brain regions and at the time-points studied in Figure 4, we used high magnification microscopy analysis. The initial morphology of MW8-positive aggregates were different from ubiquitin-positive inclusions, in that only nucleation centres of Htt aggregates were ubiquitinated (Fig. 7). Interestingly, MW8-immunolabelled Htt protein associated with the aggregate but not localised to the nucleation centre was not ubiquitinated (Fig. 7). It is possible that antigen sites on protein in parts of the aggregates may be masked by other proteins, but this seems unlikely, since the dense aggregate cores/nucleation centres were immunolabelled. It seems more likely that the MW8 antibody was labelling both aggregated and aggregating Htt, whereas ubiquitin labelled only Htt that was already aggregated. It is also notable that we could observe MW8-labelled cells before any aggregates were visible supporting the idea that there is an oligomerisation of Htt before visible aggregates form [43], [44], [45]. Again, this was not seen with ubiquitin staining, supporting our suggestion that the protein was not ubiquitinated before it aggregated.

By 41 days, neurons reach their developmental end-stage, there was complete overlap between MW8 and ubiquitin immonolabelling (Fig. 7), with ubiquitinated inclusion very similar to that of MW8-positive aggregates in all regions apart from CA3 neurons. Note, the precise timing of ubiquitination was not determined in the case of inclusions in CA3 neurons, because the aggregates in these cells develop later than in other regions. Htt aggregates are present in CA3 by 29 days of age, but the MW8 staining pattern in CA3 neurons was indistinguishable from the ubiquitin pattern, with regard to size and morphology, only by 63 days (Fig. 7).

The only region in which the staining pattern was different from that of Hf and CTX was the STR. In the STR, aggregates with clear nucleation centres appeared at around 25 days (Fig. 7). However, there was no lag in the appearance of ubiquitin-labelled inclusions, which were present in STR neurones at the same time (Fig. 7) and not at around 41 days (Fig. 5, 6). In contrast to what was seen in other regions, the ubiquitinated inclusions in the STR appeared to be much larger than the MW8-positive aggregates. We suggest that these aggregates formed a core or nucleation centre of Htt surrounded by ubiquitinated proteins that were not MW8-positive. While the size of the Htt-positive aggregates in the STR grew with time, the ubiquitinated inclusions did not appear to change much in size (Fig. 7), and the ubiquitinated inclusions remained larger than the Htt nucleation centres for several weeks. MW8-positive aggregate growth continued in the STR until 63 days, by which time STR inclusions appeared to have reached a stable state, and there was no longer an obvious morphological difference between the MW8-immunolabelled aggregates and the ubiquitin-labelled inclusions (Fig. 7).

## Discussion

Htt aggregates have not been reported in R6/2 brains before 3 weeks of age. Here, we have been able to capture an earlier time course of aggregate formation earlier than has been shown previously. We find that aggregates are already present in R6/2 brain by 2 weeks of age. Thus, visible mutant Htt aggregation in immature R6/2 brain appears at a time much earlier than previously thought. Even though it is only a week earlier than previously reported, this is likely to be important as the first 2–3 weeks of life in a mouse is the critical time for brain and synaptic development, and cells are particularly vulnerable to excitotoxic insults in the immediate postnatal period [46]. Furthermore, and in contrast to other studies where ubiquitinated inclusions are considered to be indistinguishable from Htt-positive inclusions, we show a dissociation in the pattern of aggregate formation as shown by MW8- or ubiquitin staining. From our data we conclude that the pattern of aggregation of mutant Htt is not first recognised as foreign/abnormal, ubiquitinated, and then targeted to the proteasome where aggregation occurs, but rather that the proteins aggregate and then ubiquitination occurs (or ubiquitinated protein is recruited to the aggregates).

We showed previously that inclusions form in juvenile R6/2 brain in distinct phases that consist of (i) precipitation of aggregation centres, (ii) growth of inclusions and (iii) formation of extranuclear neuronal inclusions (ENNIs) [25]. Our previous investigations focused solely on ubiquitinated inclusions, which appear precipitously at around 3 weeks. A more recent immunostaining study of inclusion formation in R6/2 brains showed similar results, with identical overlapping patterns of inclusion labelling using EM48 and ubiquitin antibodies [42]. Our results generally agree with these of Meade *et al*., [42] and support the present model of aggregate formation in which nucleation centres seed further aggregation of mutant proteins [41]. However, the EM48 staining done by Meade *et al.* failed to label any aggregates that were not ubiquitinated at any point in the time-course, and they describe the two immunostaining patterns as identical [42]. This discrepancy might be due to the fact that although both MW8 and EM48 bind to epitopes of the C-ter region, MW8 selectively binds aggregated Htt and is therefore more selective to the mutant form of the protein [38], [47].

### Aggregate Formation is a Very Rapid Event that is Differentially Timed According to Brain Region

Most prior studies, including our own, have never observed the rapidity with which the early events occur. This is because in most studies tissue is collected at weekly or even monthly intervals. Our tissue collection protocol allowed us to see changes that happened within 24 hours. We found that *in vivo,* visible aggregate formation is rapid, occurring within 24 hours. Interestingly, this parallels the situation we observed earlier with aggregation in tissue culture, where inclusions typically take 15–30 hours to form [22]. Protein aggregation has been described as a nucleation-dependent process [48]. Nucleated growth polymerization starts with the energetically unfavourable formation of a nucleus (i.e., nucleation), followed by efficient elongation of the nucleus via sequential additions of monomers [49], [50]. In spite of the fact that nucleation is considered to be a slow process, and has been shown to be roughly 1,000 times slower in the cell than *in vitro* [51]. However, we show here that in most of the cells, Htt aggregates (visualised with MW8) appeared almost overnight.

### Aggregates Develop Late in the Striatum

In addition to being precisely timed, aggregate formation was found to be brain region-dependent. Aggregates formed first in the IG, then in the CA1, subsequently in the CTX, followed by STR and finally in the DG and CA3. It is not clear what determines the pattern of aggregate formation, but it is unlikely to be correlated with neuronal vulnerability [52]. Nor does it relate to the putative vulnerability of neurons, since compared to the cortex and hippocampus, the appearance of aggregate formation in the STR, the region most affected by neurodegeneration in HD [52], [53], lagged behind that in other regions. There are of course a number of different consequences of late development of aggregates in the striatum. If the aggregates are toxic, the early aggregate formation in cortical neurons could result in abnormal synaptic input to the striatum. There is ample and elegant evidence for early corticostriatal dysfunction in R6/2 mice [54], [55], [56], [57]. On the other hand, if the aggregates are protective, the fact that aggregation of Htt in the striatal cells is delayed may make them more vulnerable to the toxicity of non-aggregated Htt [17]. Why there is such a big difference in the time-course of aggregation in the striatum compared the rest of the brain is also of particular interest, given that the striatum is most vulnerable to neurodegeneration. It is not possible to determine neuronal function or dysfunction from histopathological observations. In our opinion, it is likely that aggregate formation has complex downstream consequences that may be both beneficial and deleterious depending on the time and age of the animals [22], [25].

It is important to stress that all transgenic mouse models of HD develop neuronal inclusions. In R6/2 mice NIIs develop prior to the onset of neurological symptoms [2], [7]. By contrast, in N171-82Q and in YAC128 mice NIIs appear after the symptoms [58], [59]. Finally in BACHHD mice inclusions are mainly (>90%) extranuclear [60]. Interestingly, the presence of aggregates is not more prevalent in neuronal subpopulations known to die earliest in HD, nor does it seem to correlate to neuronal death [16], [17], [59], [60]. Therefore, despite studies suggesting a causal relationship, it is still not clear if aggregates play an active role in HD pathogenesis, or if they are simply markers of pathology. More studies about aggregate formation are needed.

### Ubiquitination Occurs after Aggregate Formation

Our results agree with those of Meade *et al*., where no ubiquitinated inclusions were found at all in CTX at 2 weeks (but were present in all layers by 3.5 weeks). We found that the time that elapsed between Htt aggregation and inclusion ubiquitination was dependent of the soma size, with a longer lag between initial aggregate formation and ubiquitination for large cells.

Our results support previous observations that ubiquitin associates at a relatively late stage in the maturation of aggregates into inclusions [62], [63]. Although we [30] and several other groups have reported occasional aggregates [2], [16], [61] and microaggregates [16], [61] that were not ubiquitinated, none of these have examined this in detail, and none have used tissue taken from sort interval timecourses that would allow a detailed study of this observation. In our previous study [30] we used PC12 cells to track aggregate morphology using time-lapse microscopy. We found clear evidence for aggregates that were not ubiquitinated at early stages of formation. Nevertheless, it was not clear if this phenomenon would be observed in brain tissue as well as immortalized cell lines. We have now shown that this appears to be present in the brain of R6/2 mice.

Given the early timing of the events we have reported (when aggregates are just beginning to appear), and the fact that we did not see aggregates that were partially ubiquitinated, we have interpreted our data as being due to the fact that ubiquitination of the protein in the aggregates has not yet occurred, or if it has, it is not enough to be visualised. It should be noted however that the presence of non-ubiquitinated aggregates could be due to de-ubiquitination of mutant htt. This is an interesting possibility, although testing it is beyond the scope of the current study, since the dynamics of ubiquitination/de-ubuqitination cannot be measured in post mortem tissue. Since there was little to no increase in background ubiquitin staining with the appearance of inclusions, in contrast to the increase in background expression of MW8-positive staining (data not shown), the most parsimonious explanation for our data is that monomeric mutant Htt is not recognised as ‘toxic’ until aggregation has started. We cannot rule out the possibility that soluble Htt might be ubiquitinated. However, in the R6/2 mice there is only normal full length Htt, so it seems unlikely that it will contribute to the pathological process at the earliest stages of aggregate formation.

We do not know the reason for the ubiquitination. It is possible that it is only after Htt has started to aggregate that it is recognised as foreign and ubiquitinated. However, importantly, to our knowledge, it has never been shown directly that mutant Htt is ubiquitinated. It has been assumed that mutant huntingtin is ubiquitinated and targeted for degradation by the ubiquitin-proteasome system pathway, because mutant huntingtin is an abnormal moiety found in neuronal intranuclear inclusions, neuronal nuclear inclusions are ubiquitinated and proteasome fragments are associated with NIIs. The possibility that mutant Htt is not recognised as foreign is consistent with the late onset of the disease in humans, where there is very little pathology for 30–40 years in the case of most adults, despite expression from both alleles [64], [65], [66], [67], [68], [69]. Another possible explanation for our finding is that ubiquitin does not label mutant Htt but labels other proteins whose recruitment and inactivation trigger detrimental effects. Mutant Htt is known to recruit WT Htt via its polyglutamine tract, and normal Htt has been found to associate with an ubiquitin-conjugating enzyme (hE2-25K) in a manner that is not modulated by the length of the polyglutamine stretch [70]. Since normal Htt is larger and has more ubiquitination sites than the mutant fragments expressed in R6/2 brain [71]. It is possible that the normal Htt, rather than mutant Htt, may be the target of ubiquitination in R6/2 brain. It is also possible that proteins other than Htt containing a poly(Gln) sequence are ubiquitinated and recruited during aggregate formation [72], [73], [74], [75], [76].

### Conclusion

Our results show for the first time that Htt aggregation in mouse brain is not only an early event, but that it occurs rapidly. Furthermore, ubiquitination is temporally dissociated from Htt aggregation. Both the timing of inclusion formation and the cellular recognition of the mutant protein as toxic are likely to be important events in HD pathology. The aggregation events we describe appear very early in R6/2 brain, well before any obvious detectable neurological phenotype. The onset of symptoms may signal a change in the role of aggregates from cytoprotective, to neurotoxic.

There are many controversial theories about pathological mechanisms in HD and their link with inclusion formation. However, Htt aggregation precedes symptoms in patients and mouse models. Therefore, characterizing the early stages of aggregate formation in mouse models are important steps that may help us understand the relationship between inclusion formation and mechanisms underlying cell death in HD.

## References

[1] The Huntington’s Disease Collaborative Research Group (1993). A novel gene containing a trinucleotide repeat that is expanded and unstable on Huntington’s disease chromosomes.. Cell.

[2] Davies SW, Turmaine M, Cozens BA, DiFiglia M, Sharp AH (1997). Formation of neuronal intranuclear inclusions underlies the neurological dysfunction in mice transgenic for the HD mutation.. Cell.

[3] Roizin L, Stellar S, Liu JC (1979). Neuronal nuclear-cytoplasmic changes in Huntington’s chorea: electron microscope investigations. In Chase TN, Wexler NS and Barbeau A, eds. *Advances in Neurology, Huntington’s Disease*.. New York, NY: Raven Press 95–122.

[4] DiFiglia M, Sapp E, Chase KO, Davies SW, Bates GP (1997). Aggregation of huntingtin in neuronal intranuclear inclusions and dystrophic neurites in brain.. Science.

[5] Yamada M, Sato T, Tsuji S, Takahashi H (2008). CAG repeat disorder models and human neuropathology: similarities and differences.. Acta Neuropathol.

[6] Gil JM, Rego AC (2009). The R6 lines of transgenic mice: a model for screening new therapies for Huntington’s disease.. Brain Res Rev.

[7] Ordway JM, Tallaksen-Greene S, Gutekunst CA, Bernstein EM, Cearley JA (1997). Ectopically expressed CAG repeats cause intranuclear inclusions and a progressive late onset neurological phenotype in the mouse.. Cell.

[8] Cooper JK, Schilling G, Peters MF, Herring WJ, Sharp AH (1998). Truncated N-terminal fragments of huntingtin with expanded glutamine repeats form nuclear and cytoplasmic aggregates in cell culture.. Hum Mol Genet.

[9] Rubinsztein DC, Wyttenbach A, Rankin J (1999). Intracellular inclusions, pathological markers in diseases caused by expanded polyglutamine tracts?. J Med Genet.

[10] Rankin J, Wyttenbach A, Rubinsztein DC (2003). Intracellular green fluorescent protein-polyalanine aggregates are associated with cell death.. Biochem J.

[11] Sánchez I, Mahlke C, Yuan J (2003). Pivotal role of oligomerization in expanded polyglutamine neurodegenerative disorders.. Nature.

[12] Tanaka M, Machida Y, Niu S, Ikeda T, Jana NR (2004). Trehalose alleviates polyglutamine-mediated pathology in a mouse model of Huntington disease.. Nat Med.

[13] Chopra V, Fox JH, Lieberman G, Dorsey K, Matson W, et al. ()2007 A small-molecule therapeutic lead for Huntington’s disease: preclinical pharmacology and efficacy of C2–8 in the R6/2 transgenic mouse.. Proc Natl Acad Sci USA.

[14] Masuda N, Peng Q, Li Q, Jiang M, Liang Y (2008). Tiagabine is neuroprotective in the N171–82Q and R6/2 mouse models of Huntington’s disease.. Neurobiol Dis.

[15] Saudou F, Finkbeiner S, Devys D, Greenberg ME (1998). Huntingtin acts in the nucleus to induce apoptosis but death does not correlate with the formation of intranuclear inclusions.. Cell.

[16] Gutekunst CA, Li SH, Yi H, Mulroy JS, Kuemmerle S (1999). Nuclear and neuropil aggregates in Huntington’s disease: relationship to neuropathology.. J Neurosci.

[17] Kuemmerle S, Gutekunst CA, Klein AM, Li XJ, Li SH (1999). Huntington aggregates may not predict neuronal death in Huntington’s disease.. Ann Neurol.

[18] Hansson O, Guatteo E, Mercuri NB, Bernardi G, Li XJ (2001). Resistance to NMDA toxicity correlates with appearance of nuclear inclusions, behavioural deficits and changes in calcium homeostasis in mice transgenic for exon 1 of the huntington gene.. Eur J Neurosci.

[19] Arrasate M, Mitra S, Schweitzer ES, Segal MR, Finkbeiner S (2004). Inclusion body formation reduces levels of mutant huntingtin and the risk of neuronal death.. Nature.

[20] Slow EJ, Graham RK, Osmand AP, Devon RS, Lu G (2005). Absence of behavioral abnormalities and neurodegeneration in vivo despite widespread neuronal huntingtin inclusions.. Proc Natl Acad Sci USA.

[21] Bodner RA, Outeiro TF, Altmann S, Maxwell MM, Cho SH (2006). Pharmacological promotion of inclusion formation: a therapeutic approach for Huntington’s and Parkinson’s diseases.. Proc Natl Acad Sci USA.

[22] Gong B, Lim MC, Wanderer J, Wyttenbach A, Morton AJ (2008). Time-lapse analysis of aggregate formation in an inducible PC12 cell model of Huntington’s disease reveals time-dependent aggregate formation that transiently delays cell death.. Brain Res Bull.

[23] Zuchner T, Brundin P (2008). Mutant huntingtin can paradoxically protect neurons from death.. Cell Death Differ.

[24] Morton AJ, Glynn D, Leavens W, Zheng Z, Faull RL (2009). Paradoxical delay in the onset of disease caused by super-long CAG repeat expansions in R6/2 mice.. Neurobiol Dis.

[25] Morton AJ, Lagan MA, Skepper JN, Dunnett SB (2000). Progressive formation of inclusions in the striatum and hippocampus of mice transgenic for the human Huntington’s disease mutation.. J Neurocytol.

[26] Maat-Schieman M, Roos R, Losekoot M, Dorsman J, Welling-Graafland C (2007). Neuronal intranuclear and neuropil inclusions for pathological assessment of Huntington’s disease.. Brain Pathol.

[27] Martindale D, Hackam A, Wieczorek A, Ellerby L, Wellington C (1998). Length of huntingtin and its polyglutamine tract influences localization and frequency of intracellular aggregates.. Nat Genet.

[28] Herndon ES, Hladik CL, Shang P, Burns DK, Raisanen J (2009). Neuroanatomic profile of polyglutamine immunoreactivity in Huntington disease brains.. J Neuropathol Exp Neurol.

[29] de Pril R, Fischer DF, Maat-Schieman ML, Hobo B, de Vos RA (2004). Accumulation of aberrant ubiquitin induces aggregate formation and cell death in polyglutamine diseases.. Hum Mol Genet.

[30] Wanderer J, Morton AJ (2007). Differential morphology and composition of inclusions in the R6/2 mouse and PC12 cell models of Huntington’s disease.. Histochem Cell Biol.

[31] Schwab C, Arai T, Hasegawa M, Yu S, McGeer PL (2008). Colocalization of transactivation-responsive DNA-binding protein 43 and huntingtin in inclusions of Huntington disease.. J Neuropathol Exp Neurol.

[32] Mangiarini L, Sathasivam K, Seller M, Cozens B, Harper A (1996). Exon 1 of the HD gene with an expanded CAG repeat is sufficient to cause a progressive neurological phenotype in transgenic mice.. Cell.

[33] Carter RJ, Lione LA, Humby T, Mangiarini L, Mahal A (1999). Characterization of progressive motor deficits in mice transgenic for the human Huntington’s disease mutation.. J Neurosci.

[34] Lione LA, Carter RJ, Hunt MJ, Bates GP, Morton AJ (1999). discrimination learning impairments in mice expressing the human Huntington’s disease mutation.. J Neurosci.

[35] Murphy KP, Carter RJ, Lione LA, Mangiarini L, Mahal A (2000). Abnormal synaptic plasticity and impaired spatial cognition in mice transgenic for exon 1 of the human Huntington’s disease mutation.. J Neurosci.

[36] Gibson HE, Reim K, Brose N, Morton AJ, Jones S (2005). A similar impairment in CA3 mossy fibre LTP in the R6/2 mouse model of Huntington’s disease and in the complexin II knockout mouse.. Eur J Neurosci.

[37] Bibb JA, Yan Z, Svenningsson P, Snyder GL, Pieribone VA (2000). Severe deficiencies in dopamine signaling in presymptomatic Huntington’s disease mice.. Proc Natl Acad Sci USA.

[38] Ko J, Ou S, Patterson PH (2001). New anti-huntingtin monoclonal antibodies: implications for huntingtin conformation and its binding proteins.. Brain Res Bull.

[39] Landles C, Sathasivam K, Weiss A, Woodman B, Moffitt H (2010). Proteolysis of mutant huntingtin produces an exon 1 fragment that accumulates as an aggregated protein in neuronal nuclei in Huntington disease.. J Biol Chem.

[40] Miller J, Arrasate M, Shaby BA, Mitra S, Masliah E (2010). Quantitative relationships between huntingtin levels, polyglutamine length, inclusion body formation, and neuronal death provide novel insight into huntington’s disease molecular pathogenesis.. J Neurosci.

[41] Chen S, Berthelier V, Yang W, Wetzel R (2001). Polyglutamine aggregation behavior in vitro supports a recruitment mechanism of cytotoxicity.. J Mol Biol.

[42] Meade CA, Deng YP, Fusco FR, Del Mar N, Hersch S (2002). Cellular localization and development of neuronal intranuclear inclusions in striatal and cortical neurons in R6/2 transgenic mice.. J Comp Neurol.

[43] Cajavec B, Bernard S, Herzel H (2005). Aggregation in Huntington’s disease: insights through modelling.. Genome Inform.

[44] Poirier MA, Jiang H, Ross CA (2005). A structure-based analysis of huntingtin mutant polyglutamine aggregation and toxicity: evidence for a compact beta-sheet structure.. Hum Mol Genet.

[45] Zhang QC, Yeh TL, Leyva A, Frank LG, Miller J (2011). A compact beta model of huntingtin toxicity.. J Biol Chem.

[46] Bhide PG, Day M, Sapp E, Schwarz C, Sheth A (1996). Expression of normal and mutant huntingtin in the developing brain.. J Neurosci.

[47] Southwell AL, Patterson PH (2010). Antibody therapy in neurodegenerative disease.. Rev Neurosci.

[48] Koo EH, Lansbury PT, Kelly JW (1999). Amyloid diseases: Abnormal protein aggregation in neurodegeneration.. Proc Natl Acad Sci USA.

[49] Ferrone F (1999). Analysis of protein aggregation kinetics.. Methods Enzymol.

[50] Wetzel R (2006). Nucleation of huntingtin aggregation in cells.. Nat Chem Biol.

[51] Colby DW, Cassady JP, Lin GC, Ingram VM, Wittrup KD (2006). Stochastic kinetics of intracellular huntingtin aggregate formation.. Nat Chem Biol.

[52] Fusco FR, Chen Q, Lamoreaux WJ, Figueredo-Cardenas G, Jiao Y (1999). Cellular localization of huntingtin in striatal and cortical neurons in rats: lack of correlation with neuronal vulnerability in Huntington’s disease.. J Neurosci.

[53] Sharp AH, Loev SJ, Schilling G, Li SH, Li XJ (1995). Widespread expression of Huntington’s disease gene (IT15) protein product.. Neuron.

[54] Kung VW, Hassam R, Morton AJ, Jones S (2007). Dopamine-dependent long term potentiation in the dorsal striatum is reduced in the R6/2 mouse model of Huntington’s disease.. Neuroscience.

[55] Cepeda C, Hurst RS, Calvert CR, Hernández-Echeagaray E, Nguyen OK (2003). Transient and progressive electrophysiological alterations in the corticostriatal pathway in a mouse model of Huntington’s disease.. J Neurosci.

[56] Cummings DM, André VM, Uzgil BO, Gee SM, Fisher YE (2009). Alterations in cortical excitation and inhibition in genetic mouse models of Huntington’s disease.. J Neurosci.

[57] André VM, Cepeda C, Fisher YE, Huynh M, Bardakjian N (2011). Differential electrophysiological changes in striatal output neurons in Huntington’s disease.. J Neurosci.

[58] Gardian G, Browne SE, Choi DK, Klivenyi P, Gregorio J (2005). Neuroprotective effects of phenylbutyrate in the N171–82Q transgenic mouse model of Huntington’s disease.. J Biol Chem.

[59] Slow EJ, van Raamsdonk J, Rogers D, Coleman SH, Graham RK (2003). Selective striatal neuronal loss in a YAC128 mouse model of Huntington disease.. Hum Mol Genet.

[60] Gray M, Shirasaki DI, Cepeda C, André VM, Wilburn B (2008). Full-length human mutant huntingtin with a stable polyglutamine repeat can elicit progressive and selective neuropathogenesis in BACHD mice. J Neurosci..

[61] Menalled LB, Sison JD, Wu Y, Olivieri M, Li XJ (2002). Early motor dysfunction and striosomal distribution of huntingtin microaggregates in Huntington’s disease knock-in mice.. J Neurosci.

[62] Senut MC, Suhr ST, Kaspar B, Gage FH (2000). Intraneuronal aggregate formation and cell death after viral expression of expanded polyglutamine tracts in the adult rat brain.. J Neurosci.

[63] Wyttenbach A, Swartz J, Kita H, Thykjaer T, Carmichael J (2001). Polyglutamine expansions cause decreased CRE-mediated transcription and early gene expression changes prior to cell death in an inducible cell model of Huntington’s disease.. Hum Mol Genet.

[64] Wexler NS, Young AB, Tanzi RE, Travers H, Starosta-Rubinstein S (1987). Homozygotes for Huntington’s disease.. Nature.

[65] Myers RH, Leavitt J, Farrer LA, Jagadeesh J, McFarlane H (1989). Homozygote for Huntington disease.. Am J Hum Genet.

[66] Kremer B, Goldberg P, Andrew SE, Theilmann J, Telenius H (1994). A worldwide study of the Huntington’s disease mutation.. N Engl J Med.

[67] Durr A, Hahn-Barma V, Brice A, Pêcheux C, Dodé C (1999). Homozygosity in Huntington’s disease.. J Med Genet.

[68] Laccone F, Engel U, Holinski-Feder E, Weigell-Weber M, Marczinek K (1999). DNA analysis of Huntington’s disease. Five years of experience in Germany, Austria, and Switzerland.. Neurology.

[69] Squitieri F, Gellera C, Cannella M, Mariotti C, Cislaghi G (2003). Homozygosity for CAG mutation in Huntington disease is associated with a more severe clinical course.. Brain.

[70] Kalchman MA, Graham RK, Xia G, Koide HB, Hodgson JG (1996). Huntingtin is ubiquitinated and interacts with a specific ubiquitin-conjugating enzyme.. J Biol Chem.

[71] Bennett EJ, Shaler TA, Woodman B, Ryu KY, Zaitseva TS (2007). Global changes to the ubiquitin system in Huntington’s disease.. Nature.

[72] Huang CC, Faber PW, Persichetti F, Mittal V, Vonsattel JP (1998). Amyloid formation by mutant huntingtin: threshold, progressivity and recruitment of normal polyglutamine proteins.. Somat Cell Mol Genet.

[73] Kazantsev A, Preisinger E, Dranovsky A, Goldgaber D, Housman D (1999). Insoluble detergent-resistant aggregates form between pathological and nonpathological lengths of polyglutamine in mammalian cells.. Proc Natl Acad Sci USA.

[74] Steffan JS, Bodai L, Pallos J, Poelman M, McCampbell A (2001). Histone deacetylase inhibitors arrest polyglutamine-dependent neurodegeneration in Drosophila.. Nature.

[75] McCampbell A, Taylor JP, Taye AA, Robitschek J, Li M (2000). CREB-binding protein sequestration by expanded polyglutamine.. Hum Mol Genet.

[76] Nucifora FC, Sasaki M, Peters MF, Huang H, Cooper JK (2001). Interference by huntingtin and atrophin-1 with cbp-mediated transcription leading to cellular toxicity.. Science.

